# Supplementary material for: Anxiety and Depression in Belgium during the First 15 Months of the COVID-19 Pandemic: A Longitudinal Study
Source: Behav Sci (Basel). 2022 May 12;12(5):141. doi: 10.3390/bs12050141 (PMC9137576; doi:10.3390/bs12050141)
Supplement: Supplementary file 1 [file behavsci-12-00141-s001.zip › S1. Demographic information Belgian Health Interview Survey 2018.pdf]

Supplementary Material

Table S1. Demographic information Belgian Health Interview Survey 2018

		N	%
Gender	Men	5588	48.1
	Women	6023	51.9
Region	Flanders	4296	37.0
	Brussels	3099	26.7
	Walloon	4216	36.3
Age	18-29	1387	14.7
	30-49	3124	33.1
	50-64	2545	27.0
	65+	2383	25.2
Average age ± standard deviation		43.03 ± 23.7	
Household composition	Single	2151	15.5
	One parent with child(ren)	1276	11.0
	Couple without child(ren)	2598	22.4
	Couple with child(ren)	5017	43.2
	Other or unknown	569	4.9
Work status	Paid job	7643	65.87
	Unemployed	386	3.3
	Sick or disabled	470	4.1
	Student	10	0.09
	Retirement	2497	21.5
	I do the housekeeping, without benefits	385	3.3
	I am a family worker	12	0.10
	Other	201	1.73
Education	Secondary degree (or lower)	2377	27.4
	Higher degree	6301	72.6
