# Supplementary material for: Anxiety and Depression in Belgium during the First 15 Months of the COVID-19 Pandemic: A Longitudinal Study
Source: Behav Sci (Basel). 2022 May 12;12(5):141. doi: 10.3390/bs12050141 (PMC9137576; doi:10.3390/bs12050141)
Supplement: Supplementary file 1 [file behavsci-12-00141-s001.zip › S2. Descriptive analysis missing data.pdf]

Supplementary Material

Table S2. Descriptive analysis missing data

Variable	N missing	%Missing
Anxiety (total score)	75	0.68%
Depression (total score)	119	1.08%
Gender	53	0.5%
Region	79	0.72%
Age	224	2.03%
Household composition	38	0.34%
Work status	498	4.5%
Education	53	0.5%
Social support	712	6.46%
Exposure COVID-19	30	0.27%
Appointment Psychologist/psychotherapist/psychiatrist	720	6.53%
Personality trait	306	2.78%
